# Evaluation of a Family-Based, Health Literacy-Adapted Educational Intervention Program in Patients With Type 2 Diabetes Mellitus

**DOI:** 10.7759/cureus.80239

**Published:** 2025-03-07

**Authors:** Panagiotis Panagiotidis, Athena Kalokairinou, Chara Tzavara, Anastasia Michailidou, Venetia-Sofia Velonaki

**Affiliations:** 1 Nursing Department, National and Kapodistrian University of Athens, Athens, GRC; 2 Outpatient Diabetes Clinic, General Hospital of Drama, Drama, GRC; 3 Home Nursing Department, General Hospital of Drama, Drama, GRC; 4 Biostatistics Department, National and Kapodistrian University of Athens, Athens, GRC

**Keywords:** diabetes education, diabetes type 2, glycemic control, health literacy, home-based education

## Abstract

Background

Glycemic goals are linked to both health literacy (HL) and self-efficacy (SE) in type 2 diabetes mellitus (T2DM) management. This study aims to investigate the effectiveness of an HL-adapted educational intervention for patients with type T2DM at home in achieving glycemic control goals and improving HL and SE.

Method

This randomized controlled trial involved an intervention group and a control group, comprising random samples of 60 patients with T2DM monitored at the diabetes clinic and home care department of the Hospital of Drama, Drama, Greece. The intervention group participated in a structured home education program, while the control group continued to receive standard care during routine visits. All participants completed the following two scales at baseline, immediately post-intervention, and three months after intervention: the short form of the European Health Literacy Survey Questionnaire (HLS-EU-Q16) to measure HL and the Diabetes Management Self-Efficacy Scale (DMSES) to measure SE. Demographic characteristics, BMI, medical history, and glycemic control metrics, including HbA1c (A1C), fasting plasma glucose (FPG), and postprandial plasma glucose (PPG) were recorded.

Results

We approached 130 T2DM patients, of whom 120 participated (92.3% response rate), evenly split between intervention and control groups. The groups had similar baseline characteristics. Three months post-intervention, the intervention group showed significant improvements in glycemic control (A1C, FPG, PPG), weight, HL, and SE. The proportion of patients achieving glycemic goals increased substantially in the intervention group. SE fully mediated the relationship between HL and A1C immediately after the intervention. Overall, the intervention group demonstrated superior outcomes compared to the control group.

Conclusion

The findings confirm that family-based and HL-adapted intervention programs can effectively support the management of T2DM. Such interventions can help patients achieve glycemic control goals while enhancing HL and SE.

## Introduction

Diabetes mellitus (DM) is a chronic disease with an increasing incidence worldwide [1]. Its complications impact patients’ quality of life and place a financial burden on patients, families, and health systems. In 2021, an estimated 529 million individuals worldwide had DM, a number projected to more than double in 2050 [1]. In Greece, the overall prevalence of DM (both diagnosed and undiagnosed) is 11.9% [2], with approximately 96% of cases being type 2 diabetes mellitus (T2DM) [1].

According to the American Diabetes Association, achieving glycemic targets is critical in preventing complications. For most non-pregnant patients, these targets include HbA1c (A1C) <7%, fasting plasma glucose (FPG) level of 80-130 mg/dL, and postprandial plasma glucose (PPG) level of <180 mg/dL [3].

Despite advances in technology and new self-monitoring devices and medications, only 42.8% of T2DM patients meet A1C targets [4]. Tailored educational interventions focusing on glucose monitoring, meal planning, medication adherence, physical activity, foot control, and stress management may be key to improving glycemic control [5].

Both individual and group education programs for T2DM patients lead to important patient outcomes, although these effects seemed to diminish over time post-intervention was completed [6].

Health literacy (HL) is a key social determinant of health, directly influencing patients' ability to manage chronic conditions effectively [7]. Higher HL levels are associated with improved DM self-management and better glycemic control [8]. Conversely, inadequate HL poses a significant barrier to developing essential DM self-management skills [8].

Self-efficacy (SE) is a fundamental psychological construct that reflects an individual's belief in their ability to organize and execute actions required to achieve specific goals [9]. In the context of DM management, SE plays a crucial role in fostering confidence in self-care behaviors, influencing patients' ability to adhere to treatment regimens and maintain glycemic control.

Population-based studies confirm the positive association between HL, SE, and glycemic control, with SE mediating the relationship between HL and A1C [8,10]. Moreover, culturally sensitive and family-supported DM education programs further strengthen these outcomes, particularly in patients with low HL [11]. Family-focused interventions enhance SE, self-management, and glycemic control, making them especially effective in culturally familial societies [12].

To our knowledge, few studies have evaluated the effectiveness of interventions tailored to the HL of patients with T2DM. Even fewer have been conducted in patients' homes with the involvement of their family environment, and none has been conducted in Greece. This gap is particularly significant as HL and family support are crucial for improving self-care adherence and glycemic control, especially in culturally familial societies where family plays a central role in health-related decision-making. This study aims to evaluate the effectiveness of a home-based education program for patients with T2DM, involving family and/or caregivers and tailored to the patients' HL, in achieving glycemic goals (A1C, FPG, and PPG) and improving HL and SE.

## Materials and methods

Research design

This is a randomized controlled trial comprising an intervention group and a control group, with data collection at three time points: before the intervention, immediately after the intervention, and three months post-intervention.

To calculate the sample size, power analysis was performed with two levels of comparisons between groups (intervention and control) and two levels of comparisons over time (analysis of variance for repeated comparisons).

To ensure the required random sample of 120 patients (n=60 control group and n=60 intervention group), we had to approach 130 people, of which 10 refused to participate in the study (RR= 92.30%). Patient enrolment and initial data collection took approximately six months (July to December 2021). The intervention and data collection immediately after and three months post-intervention lasted approximately one year (January to December 2022). There were no dropouts during the study. The study was conducted at the Diabetes Outpatient Clinic (DOC) of Hospital of Drama, Drama, Greece, in collaboration with the hospital's home care department. The intervention was carried out at patients' homes.

Sampling strategy

The DOC of the Hospital of Drama receives approximately 3.000 visits annually from 800 patients with T2DM per year. To select the 120 patients, a lottery system was used to draw numbers from the entire sample, with separate selections for males and females. This was followed by a review to ensure that the inclusion criteria were met and that the selected individuals consented to participate. Any shortfall in the required number of participants was addressed through a supplementary draw.

Patients were randomly assigned to either the control or intervention group through a lottery system, which involved categorization by sex, age, disease duration, and educational level to ensure balance between the groups. A researcher not involved in the intervention performed the lottery manually. This process ensured randomization and minimized selection bias.

Inclusion Criteria

Men and women with T2DM, who permanently resided in the city of Drama and in lowland villages of the prefecture, were followed up at the DOC, had family and/or caregivers, and possessed a sufficient ability to understand basic instructions, were eligible to participate in the study.

Exclusion Criteria

Patients with psychiatric or mental illnesses, disabilities, or neoplasms or other chronic diseases with a life expectancy of less than five years were excluded. Additionally, individuals without family or caregivers, as well as those who did not provide consent to participate, were not included in the study.

Control group and intervention group: the intervention

Participants in the intervention group attended a structured training program lasting 2 hours per session. Patients receiving oral medication or non-insulin injectable treatments attended three sessions, while those receiving insulin attended four sessions. In cases where more than one person from the same family participated in the study and received different treatment (e.g., one on insulin and another on anti-diabetic tablets or other injectable treatments), four sessions were conducted. Each session included a minimum of three participants, comprising the patient and their family and/or caregivers.

The educational materials used included "discussion maps: learning about diabetes," [13] insulin pens, GLP-1 pens, glucose meters, and educational videos from insulin company websites. The sessions at the patients’ homes in the intervention group were conducted by a health visitor following a telephone consultation. Participants in the control group continued receiving usual care, which included regular visits to the DOC. During these visits, patients received general DM management instruction, including guidance on medication adherence. After assessing patients’ HL and SE and disease management challenges, the educational intervention was tailored to each patient’s characteristics.

More information about the intervention

"Conversation maps: learning about diabetes"

The intervention was based on the "Conversation maps: learning about diabetes," an educational program developed through collaboration between Healthy Interactions, Lilly, and International Diabetes Federation (IDF) Europe. Patient-centered and combining verbal and visual learning [13], the program is designed for groups of 3-10 participants, including people with T2DM, their families, and caregivers. It aims to change attitudes and behaviors through knowledge acquisition and interaction with the treatment team. The program consists of four modules: the first three are for all T2DM patients and the fourth is for insulin users. The modules cover 1) general DM overview, 2) how DM works, 3) healthy diet and exercise, and 4) starting insulin therapy. Each section takes about two hours to complete.

Tools

A questionnaire was used to record the participants’ demographic characteristics, comorbidities, DM complications, and A1C, FPG, and PPG levels. The researcher also measured height and weight to calculate body mass index (BMI). For the evaluation of the participants' HL and SE, they were asked to answer the Greek versions of the European Health Literacy Survey Questionnaire - 16 items (HLS-EU-Q16) [14,15] and the Diabetes Management Self-Efficacy Scale (DMSES) [16,17].

The HLS-EU-Q16 assesses the cognitive aspect of HL covering four skills: assessing, understanding, evaluating, and applying health information. Responses use a four-point Likert scale. The scale has been widely used in studies with both healthy individuals and patients with chronic conditions, including those with DM. In this study, the HLS-EU-Q16 showed acceptable reliability, with a Cronbach's alpha of 0.72.

The DMSES is a widely used tool to assess SE in patients with T2DM. It consists of 20 questions divided into four subscales: diet (9 questions), medical treatment (5 questions), medication and foot control (3 questions), and physical activity (3 questions), with responses on a five-point Likert scale. This study used the Greek version of the scale [17]. Cronbach's alpha indicated acceptable reliability for the overall scale (0.89) and subscales: Diet (0.89), medical treatment (0.73), medication and foot control (0.76), and physical activity (0.80).

Data collection process

Participants in both groups completed the Greek versions of the HLS-EU-Q16 and DMSES scales at study entry, immediately after the intervention, and three months post-intervention.

To calculate BMI, patients’ height and weight were measured at study inclusion. Weight was remeasured immediately after intervention and three months post-intervention, and comparisons were made to assess any changes over these periods.

Before and immediately after the intervention, A1C levels were obtained from the patients' medical records. Three months post-intervention, blood samples were collected at the DOC to measure A1C.

FPG and capillary PPG values at all three time points were calculated as the average of the past month's readings from self-monitoring diaries or devices used by the patients.

Ethical issues

Ethical approval for the research was graded by the ethics committees of the involved institutions. Patients meeting the entry criteria provided signed informed consent to be included in the sample. Data were pseudonymized, and confidentiality was maintained.

Statistical analysis

For the variables that were normally distributed, the means and standard deviations (SD) were used to describe them, while for those that were not normally distributed, the median and the interquartile range were used. Absolute (N) and relative (%) frequencies were used to describe categorical and ordinal variables. Pearson's χ2 test or Fisher's exact test was used to compare categorical characteristics between the two groups where necessary. For comparison of quantitative characteristics between the two groups, Student's t-test or the nonparametric Mann-Whitney test was used. The McNemar test was used to compare BMI, HL, and successful glycemic control rates (A1C<7%) of patients in each group between consecutive time points. Repeated measures analysis of variance (ANOVA) was used to test for differences in weight, BMI, A1C measurements and SE and HL between groups and over time. The above method was also used to assess whether the degree of change over time of the parameters under study was different between the two groups. Because of the asymmetry of the distributions, logarithmic transformations of the variables were used in the repeated measures ANOVA method. To control for type I error due to multiple comparisons, the Bonferroni correction was used, according to which the significance level is 0.05/κ (κ = number of comparisons). Linear regression analysis with the stepwise inclusion/exclusion procedure was used to find independent factors associated with SE and A1C values post-intervention, which resulted in dependence coefficients (β) and their standard errors. To investigate the correlation between changes in SE, HL and A1C values, linear mixed models were performed, from which dependence coefficients (β) and their standard errors were obtained. Because of the asymmetry of the distributions, logarithmic transformations of the variables were used in both the linear regression analysis and the linear mixed models. The mediating role of the SE between HL and A1C was tested at different time points using the PROCESS 4.2 procedure [18]. For SE and HL, the measurement taken immediately post-intervention was used, while for A1C, the measurement was taken three months post-intervention, a time point at which changes in A1C levels could be observed. Specifically, the mediating effect was tested through confidence intervals using the bootstrapping technique with 1,000 samples to ensure 95% confidence intervals for the indirect effect (a*b coefficient). If the confidence intervals do not include zero, the mediating effect is statistically significant. Furthermore, if the direct effect (c’ coefficient) is significant, the mediation is partial; if it is not significant, the mediation is complete. The significance levels are two-sided, and statistical significance was set at 0.05. The SPSS 26.0 statistical program was used for the analysis (IBM Corp., Armonk, NY).

## Results

Characteristics of the two groups

The sample consisted of 120 patients divided into control (n=60) and intervention (n=60) groups. No significant differences in characteristics were found (p>0.05), confirming group equivalence. The mean age was 64 years (SD=10.6) in the control group and 66.9 years (SD=10.6) in the intervention group. Most participants were female (55%, 51.7%), married, or cohabiting (81.7%, 90%), lived in Drama (65%, 66.7%), and had social security (85%, 90%). DM affected one family member in 61.7% of the control group and 56.7% of the intervention group. Monthly incomes below 400 Euros were reported by 45% of the control group and 40% of the intervention group. Both groups had similar demographics (p>0.05). Table 1 provides detailed demographic data.

**Table 1:** Characteristics of the control and intervention groups ‡Student's t-test. +Pearson's χ^2^ test. ++Fisher's exact test. ‡‡Mann-Whitney test. SD, standard deviation

	Control group (n=60, 50%)	Intervention group (n=60, 50%)	ρ-Value
Age, mean (SD)	64 (10.6)	66.9 (10.6)	0.136‡
Sex, n (%)			
Men	27 (45.0)	29 (48.3)	0.714+
Women	33 (55.0)	31 (51.7)	
Marital status, n (%)			
Married or cohabiting	49 (81.7)	54 (90.0)	0.191+
Unmarried or divorced or widowed	11 (18.3)	6 (10.0)	
Number of members who have DM, n (%)			
1	37 (61.7)	34 (56.7)	0.045++
2	23 (38.3)	20 (33.3)	
3	0 (0.0)	6 (10.0)	
Place of residence, n (%)			
Drama	39 (65.0)	40 (66.7)	0.617++
Lowland villages of Drama	19 (31.7)	19 (31.7)	
Kavala	0 (0.0)	1 (1.7)	
Lowland villages of Kavala	2 (3.3)	0 (0.0)	
Education level, n (%)			
Illiterate	0 (0.0)	2 (3.3)	0.348++
Primary school graduates	23 (38.3)	29 (48.3)	
High school/Greek Lykeion graduates	31 (51.7)	25 (41.7)	
Higher education/master's degree	6 (10.0)	4 (6.7)	
Social security, n (%)			
No	9 (15.0)	6 (10.0)	0.408+
Yes	51 (85.0)	54 (90.0)	
Monthly income in euros, n (%)			
<400	27 (45.0)	24 (40.0)	0.913++
401-900	22 (36.7)	26 (43.3)	
901-1,500	10 (16.7)	9 (15.0)	
>1,500	1 (1.7)	1 (1.7)	
Number of family members, median (IQR)	3 (3–3)	3 (3–3.5)	0.694‡‡

Participants in the control group had an average DM duration of 12.7 years (SD=6.6), compared to 14.6 years (SD=7.3) in the intervention group. Half of both groups had visited their physician three times in the past year for DM management. Most participants in both groups had arterial hypertension and dyslipidemia, were treating DM with tablets (60%, 50%) and had not experienced hypoglycemia in the past three months (75%, 70%). Table 2 provides absolute and relative frequencies for the participants’ medical history, showing similar distributions between both groups (p>0.05).

**Table 2:** Medical history of the control and intervention groups Student's t-test. +Pearson's χ^2^ test. ++Fisher's exact test DM, diabetes mellitus; GLP1, glucagon-like peptide 1; SD, standard deviation

	Control group	Intervention group	ρ-Value
Years you have been diagnosed with DM, mean (SD)	12.7 (6.6)	14.6 (7.3)	0.129‡
How many times have you visited your doctor for a DM check-up in the past year? n (%)			
1	5 (8.3)	2 (3.3)	0.386++
2	9 (15.0)	15 (25.0)	
3	30 (50.0)	30 (50.0)	
4	16 (26.7)	13 (21.7)	
Diagnosed with any of the following diseases? n (%)	57 (95.0)	59 (98.3)	0.309++
If so, which			
Heart failure	12 (20.0)	18 (30.0)	0.206+
Coronary artery disease	16 (26.7)	24 (40.0)	0.121+
Arterial hypertension	50 (83.3)	46 (76.7)	0.361+
Vascular stroke	1 (1.7)	1 (1.7)	>0.999++
Severe kidney disease	2 (3.3)	6 (10.0)	0.272++
Dyslipidemia	50 (83.3)	55 (91.7)	0.168+
Depression	18 (30.0)	18 (30.0)	>0.999+
Other	31 (51.7)	26 (43.3)	0.361+
Diagnosis with any of the following chronic complications of DM? n (%)	23 (38.3)	30 (50.0)	0.198+
If so, which			
Diabetic neuropathy	23 (38.3)	27 (45.0)	0.459+
Diabetic angiopathy	7 (11.7)	10 (16.7)	0.432+
Diabetic foot	1 (1.7)	2 (3.3)	>0.999++
Diabetic retinopathy	6 (10.0)	8 (13.3)	0.570+
Diabetic nephropathy	1 (1.7)	5 (8.3)	0.207++
Other	0 (0.0)	0 (0.0)	-
DM medication, n (%)			
Diet/exercise only	0 (0.0)	0 (0.0)	0.334++
Tablets	36 (60.0)	30 (50.0)	
Tablets and insulin	3 (5.0)	5 (8.3)	
Tablets and GLP1	7 (11.7)	5 (8.3)	
Tablets, GLP1, and insulin	4 (6.7)	5 (8.3)	
GLP1	1 (1.7)	1 (1.7)	
Insulin and GLP1	0 (0.0)	5 (8.3)	
One insulin	1 (1.7)	0 (0.0)	
Insulin combination	8 (13.3)	9 (15.0)	
Have you experienced hypoglycemia in the last 3 months? n (%)			
No	45 (75.0)	42 (70.0)	0.540+
Yes	15 (25.0)	18 (30.0)	
If yes, how often			
One or more times in the last week	1 (6.7)	3 (16.7)	0.768++
One or more times in the last month	3 (20.0)	4 (22.2)	
One or more times in the last three months	11 (73.3)	11 (61.1)	

No statistically significant differences (p>0.05) were found between the two groups regarding self-monitoring behavior or glucose variability. Table 3 presents data on participants' self-monitoring of blood glucose for each group separately.

**Table 3:** Results and self-monitoring behaviors between the two groups ++Fisher's exact test. +Pearson's χ^2^ test. ‡‡Mann-Whitney test. FPG, fasting plasma glucose; PPG, postprandial plasma glucose; SD, standard deviation

	Control group	Intervention group	p-Value
Do you measure your blood glucose using an individual measuring device?			
No, n (%)	1 (1.7)	1 (1.7)	>0.999++
Yes, n (%)	59 (98.3)	59 (98.3)	
If yes,			
According to the doctor's instructions, n (%)	25 (42.4)	20 (33.9)	0.542+
More times than the doctor's recommendations, n (%)	6 (10.2)	9 (15.3)	
No measurement plan (random), n (%)	28 (47.5)	30 (50.8)	
Glucose variability (FPG), mean (SD)	143.1 (32.6)	147.4 (46.8)	0.561‡‡
Glucose variability (PPG), mean (SD)	169.2 (46.9)	177.3 (59.3)	0.411‡‡

Evaluation of the intervention

Change in A1C, FPG, and PPG

Before and immediately after the intervention, A1C values were similar in both groups. At three months post-intervention, A1C values were significantly lower in the intervention group (p<0.001), while no significant changes were observed in the control group. In the intervention group, A1C significantly decreased from 7.6% immediately post-intervention to 6.96% at three months (p<0.001).

Before and immediately post-intervention, FPG values were similar in both groups. At three months, values were significantly lower in the intervention group (p=0.001). Over time, FPG decreased significantly post-intervention in both groups. However, in the intervention group, FPG further decreased significantly at three months compared to immediately post-intervention (136.2 mg/dL to 114.5 mg/dL; p<0.001).

Before and immediately after the intervention, PPG values were similar in both groups. At three months, PPG values were significantly lower in the intervention group. While the control group showed no significant change over time, the intervention group experienced a significant decrease from before to immediately post-intervention (p=0.002) and a further decrease at three months compared to immediately post-intervention (162 mg/dL to 145.3 mg/dL; p<0.001).

The degree of change over time differed significantly between the two groups for all three variables (A1C, FPG, and PPG). Patients in the intervention group showed significant decreases, while those in the control group remained stable (A1C, PPG) or had smaller decreases (FPG) throughout follow-up. Table 4 presents the A1C, FPG, and PPG values for each measurement and group.

**Table 4:** . Changes in A1C, FPG, and PPG over time between the two groups Note: the analysis was performed using logarithmic transformations ^1^P-value for between-group comparisons. ^2^P-value for between-time comparisons after Bonferroni correction. ^3^P-value from repeated measures ANOVA. Differences in change from one measurement to another between groups. A1C, glycosylated hemoglobin; FPG, fasting plasma glucose; PPG, postprandial plasma glucose; SD, standard deviation

		Before intervention		Immediately after intervention		3 months post-intervention		Change before and immediately after intervention		Change immediately after intervention and 3 months post-intervention		Ρ^3^
	Group	Mean value (SD)	Median (IQR)	Mean value (SD)	Median (IQR)	Mean value (SD)	Median (IQR)	Mean value (SD)	Ρ^2^	Mean value (SD)	Ρ^2^	
A1C	Control	7.7 (1.58)	7.2 (6.8–8.3)	7.62 (1.16)	7.4 (6.7–8.3)	7.68 (1.11)	7.4 (6.75–8.1)	-0.08 (0.72)	>0.999	0.06 (0.42)	>0.999	<0.001
	Intervention	7.62 (1.21)	7.35 (6.8–8.15)	7.6 (1.04)	7.5 (6.85–7.9)	6.96 (0.69)	6.8 (6.5–7.4)	-0.02 (0.39)	>0.999	-0.64 (0.64)	<0.001	
	Ρ^1^	0.874		0.963		<0.001						
FPG	Control	143.1 (32.6)	138 (125–153)	135.5 (27.7)	128 (114–146)	135.9 (25.5)	132 (119–149)	-7.64 (24.88)	0.037	0.4 (16.13)	>0.999	<0.001
	Intervention	147.4 (46.8)	139 (123–155)	136.2 (29.6)	133.5 (112–154)	114.5 (21.7)	111.5 (97.5–128.5)	-11.21 (26.57)	0.003	-21.72 (23.09)	<0.001	
	Ρ^1^	0.720		0.966		<0.001						
PPG	Control	169.2 (46.9)	157 (137–188)	162.2 (32.2)	162 (139–178)	161.6 (30.9)	159.5 (139–181)	-7 (29.51)	0.544	-0.63 (16.88)	>0.999	<0.001
	Intervention	177.3 (59.3)	168 (140–189)	162 (33.6)	159 (139.5–174)	145.3 (18.3)	142 (131–156)	-15.36 (32.95)	0.002	-16.67 (25.94)	<0.001	
	Ρ^1^	0.379		0.963		0.001						

**Figure 1:**
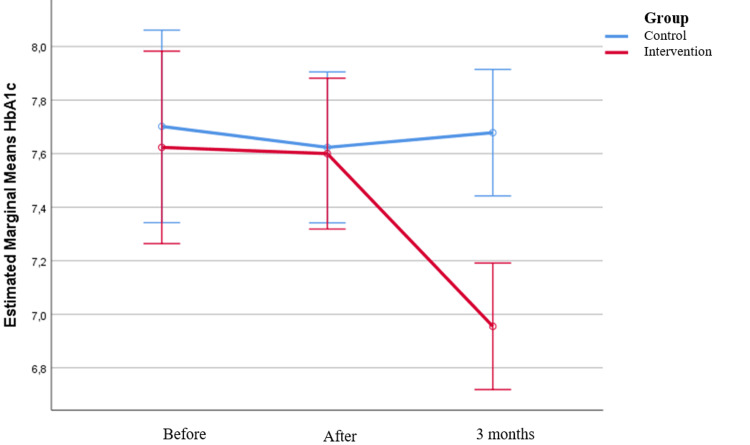
A1C changes over time in both groups HbA1C, glycosylated hemoglobin

Figure 1 shows changes in patients’ A1C over time, separately for the intervention and control groups.

The rates of achieving A1C, FPG, and PPG targets were similar between the two groups before and immediately after the intervention.

At three months post-intervention, the rate of achieving A1C goals was significantly higher in the intervention group compared to the control group (p=0.003). From before to immediately post-intervention, rates of glycemic control did not change significantly in either group. However, at three months compared to immediately post-intervention, rates changed significantly in both groups: decreasing from 40% to 30% in the control group and increasing from 28.3% to 56.7% in the intervention group.

At three months, the rate of achieving FPG goals was significantly higher in the intervention group compared to the control group (p=0.001). From before to immediately post-intervention, goal attainment rates changed significantly only in the control group. However, at three months compared to immediately post-intervention, goal attainment rates changed significantly only in the intervention group. Specifically, in the control group, the rate increased from 39% to 44.1%, while in the intervention group, it increased from 35.6% to 76.7%.

At three months, the rate of achieving PPG goals was significantly higher in the intervention group compared to the control group (p=0.005). From before to immediately post-intervention, goal attainment rates increased significantly in both groups. However, at three months compared to immediately post-intervention, goal attainment rates decreased significantly only in the control group (p<0.001). Overall, in the control group, the rate increased from 67.8% to 74.1%, while in the intervention group, it increased from 64.4% to 93.3%.

The rates of achieving A1C (<7%), FPG (80-130 mg/dL), and PPG (<180 mg/dL) targets for each measurement and group are provided in Table 5.

**Table 5:** Changes in A1C, FPG, and PPG over time in the two groups relative to goal attainment ^1^P-value for comparisons between groups via Pearson's χ^2^ test. ^2^P-value for comparisons between time measurements (McNemar test). A1C, glycosylated hemoglobin; FPG, fasting plasma glucose; PPG, postprandial plasma glucose

		Control group, n (%)	Intervention group, n (%)	Ρ^1^
Α1C				
Before intervention	<7%	23 (38.3)	22 (36.7)	0.850
	≥7%	37 (61.7)	38 (63.3)	
Immediately after intervention	<7%	24 (40)	17 (28.3)	0.178
	≥7%	36 (60)	43 (71.7)	
3 months post-intervention	<7%	18 (30)	34 (56.7)	0.003
	≥7%	42 (70)	26 (43.3)	
P^2^ before vs immediately after intervention		>0.999	0.063	
P^2 ^immediately after intervention vs 3 months post-intervention		0.031	<0.001	
FPG, mg/dL				
Before intervention	80-130	23 (39)	21 (35.6)	0.703
	<80 or >130	36 (61)	38 (64.4)	
Immediately after intervention	80-130	33 (55.9)	28 (46.7)	0.312
	<80 or >130	26 (44.1)	32 (53.3)	
3 months post-intervention	80-130	27 (46.6)	46 (76.7)	0.001
	<80 or >130	31 (53.4)	14 (23.3)	
P^2 ^before vs immediately after intervention		0.031	0.118	
P^2 ^immediately after intervention vs 3 months post-intervention		0.227	<0.001	
PPG, mg/dL				
Before intervention	<180	40 (67.8)	38 (64.4)	0.697
	>180	19 (32.2)	21 (35.6)	
Immediately after intervention	<180	59 (100)	60 (100)	-
	>180	0 (0)	0 (0)	
3 months post-intervention	<180	43 (74.1)	56 (93.3)	0.005
	>180	15 (25.9)	4 (6.7)	
P^2 ^before vs immediately after intervention		<0.001	<0.001	
P^2 ^immediately after intervention vs 3 months post-intervention		<0.001	0.125	

Correlation Between A1C Changes and Patients’ Characteristics in the Intervention Group

Throughout follow-up, patients on insulin had higher A1C values than those on other antidiabetic treatments. However, insulin-treated patients benefited more from the intervention, with the mean A1C decreasing by 0.8% three months post-intervention compared to immediately post-intervention, while those on other treatments had a 0.54% decrease.

Patients with DM-related complications had higher A1C values than those without, a difference that was not statistically significant immediately post-intervention but became significant at three months (p=0.011). Before intervention and immediately post-intervention, no A1C differences were observed based on patients' HL levels. However, at three months, while A1C decreased significantly in all (p<0.001), it was significantly lower in those with higher HL (p=0.027).

Overall, A1C significantly decreased at three months compared to immediately post-intervention in the intervention group, regardless of patient characteristics. The reduction was significantly greater in patients under 69 years of age (p=0.006) and those who had visited their physician for DM testing one to two times in the past year (p=0.029). Table 6 presents A1C changes based on the characteristics of patients in the intervention group.

**Table 6:** Changes in A1C values by the characteristics of patients in the intervention group Note: analyses were performed using logarithmic transformations ^1^P-value for between-group comparisons. ^2^P-value for between-time comparisons after Bonferroni correction. ^3^P-value from repeated measures ANOVA. Differences in change from one measurement to another between groups. A1C, glycosylated hemoglobin; HL, health literacy; SD, standard deviation

		A1C											Ρ^3^
		Before intervention		Immediately after intervention		3 months post- intervention		Change before and immediately after intervention			Change immediately after intervention 3 months post-intervention		
		Mean value (SD)	Median (IQR)	Mean value (SD)	Median (IQR)	Mean value (SD)	Median (IQR)	Mean value (SD)	Ρ^2^	Mean value (SD)		Ρ^2^	
Age	<69	7.86 (1.53)	7.4 (6.8–9)	7.82 (1.28)	7.7 (6.8–8.7)	6.94 (0.83)	7.4 (6.8–9)	-0.04 (0.48)	>0.999	-0.88 (0.74)		<0.001	0.006
	≥69	7.4 (0.77)	7.3 (6.8–8)	7.4 (0.72)	7.5 (6.9–7.8)	6.97 (0.54)	7.3 (6.8–8)	0 (0.29)	>0.999	-0.43 (0.43)		<0.001	
	Ρ^1^	0.219		0.164		0.767							
Sex	Men	7.72 (1.38)	7.6 (6.9–8.1)	7.68 (1.13)	7.5 (7–7.9)	6.9 (0.67)	7.6 (6.9–8.1)	-0.04 (0.47)	>0.999	-0.78 (0.66)		<0.001	0.155
	Women	7.54 (1.05)	7.3 (6.7–8.2)	7.53 (0.97)	7.5 (6.8–7.9)	7 (0.72)	7.3 (6.7–8.2)	-0.01 (0.31)	>0.999	-0.53 (0.59)		<0.001	
	Ρ^1^	0.624		0.595		0.591							
Monthly income (in euros)	<400	7.78 (1.55)	7.4 (6.65–8.4)	7.72 (1.35)	7.35 (6.6–8.7)	7.07 (0.87)	7.4 (6.65–8.4)	-0.06 (0.41)	>0.999	-0.65 (0.63)		<0.001	0.880
	>401	7.52 (0.93)	7.35 (6.85–8.05)	7.52 (0.78)	7.55 (7–7.8)	6.88 (0.54)	7.35 (6.85–8.05)	0 (0.38)	>0.999	-0.64 (0.65)		<0.001	
	Ρ^1^	0.563		0.628		0.365							
How many times have you visited your doctor for a DM check-up in the past year?	1-2	7.98 (1.53)	7.8 (6.8–8.9)	7.85 (1.21)	7.8 (7–8.3)	6.96 (0.76)	7.8 (6.8–8.9)	-0.13 (0.56)	0.972	-0.89 (0.87)		<0.001	0.029
	3-4	7.48 (1.04)	7.3 (6.8–8)	7.5 (0.97)	7.3 (6.8–7.8)	6.95 (0.67)	7.3 (6.8–8)	0.02 (0.29)	>0.999	-0.55 (0.5)		<0.001	
	Ρ^1^	0.180		0.270		0.979							
Insulin medication	No	7.27 (0.91)	6.95 (6.7–7.75)	7.29 (0.78)	7.15 (6.8–7.6)	6.75 (0.58)	6.95 (6.7–7.75)	0.02 (0.3)	>0.999	-0.54 (0.59)		<0.001	0.152
	Yes	8.15 (1.42)	8 (7.25–9)	8.06 (1.22)	7.8 (7.25–8.8)	7.26 (0.74)	8 (7.25–9)	-0.09 (0.49)	>0.999	-0.8 (0.68)		<0.001	
	Ρ^1^	0.005		0.005		0.005							
Diagnosis with any of the following chronic complications of DM	No	7.36 (1.01)	7.1 (6.7–8)	7.38 (0.91)	7.2 (6.8–7.8)	6.73 (0.62)	7.1 (6.7–8)	0.02 (0.32)	>0.999	-0.65 (0.63)		<0.001	0.813
	Yes	7.89 (1.35)	7.7 (6.9–8.4)	7.82 (1.13)	7.6 (7.1–8.2)	7.18 (0.69)	7.7 (6.9–8.4)	-0.07 (0.45)	>0.999	-0.64 (0.66)		<0.001	
	Ρ^1^	0.089		0.096		0.011							
HL levels (before)	Inadequate/problematic	7.73 (1.1)	7.65 (6.8–8.1)	7.65 (0.98)	7.6 (6.9–7.9)	7.17 (0.62)	7.65 (6.8–8.1)	-0.08 (0.31)	>0.999	-0.48 (0.63)		<0.001	0.112
	Sufficient	7.54 (1.3)	7.25 (6.7–8.2)	7.56 (1.1)	7.3 (6.8–8.2)	6.79 (0.7)	7.25 (6.7–8.2)	0.02 (0.44)	>0.999	-0.77 (0.62)		<0.001	
	Ρ^1^	0.458		0.669		0.027							

Weight Change and BMI

There was no significant change in weight or BMI from before intervention to immediately post-intervention in either group. However, at three months post-intervention, patients in the intervention group showed a significant decrease in weight (-2.5 kg/m^2^, p<0.001) and BMI (-0.9 kg/m^2^, p<0.001) compared to immediately post-intervention, with no significant change in the control group. The degree of change in both measures differed significantly between the groups, with the intervention group showing decreases and the control group remaining stable throughout follow-up. Table 7 presents the changes in weight and BMI.

**Table 7:** Changes in weight and BMI in the two groups ^1^P-value for between-group comparisons. ^2^P-value for between-time comparisons after Bonferroni correction. ^3^P-value from repeated measures ANOVA. Differences in change from one measurement to another between groups. SD, standard deviation

	Group	Before intervention	Immediately after intervention	3 months post-intervention	Change before intervention and immediately after intervention		Change immediately after intervention and 3 months post-intervention		Ρ^3^
		Mean value (SD)	Mean value (SD)	Mean value (SD)	Mean value (SD)	Ρ^2^	Mean value (SD)	Ρ^2^	
Body mass index	Control	32.7 (6.1)	32.7 (6.2)	32.7 (6.2)	0 (0.7)	>0.999	0 (0.5)	>0.999	<0.001
	Intervention	34 (6.6)	33.9 (6.6)	33.1 (6.1)	-0.1 (0.5)	>0.999	-0.9 (1)	<0.001	
	Ρ^1^	0.265	0.299	0.769					
Weight (in kilograms)	Control	92.6 (21)	92.6 (21.3)	92.7 (21)	0 (2)	>0.999	0.1 (1.4)	>0.999	<0.001
	Intervention	95.5 (20.4)	95.4 (20.5)	92.9 (18.8)	-0.1 (1.4)	>0.999	-2.5 (3)	<0.001	
	Ρ^1^	0.447	0.474	0.959					

SE (DMSES Scale and Subscales)

Before the intervention, the scores on the DMSES scale and its subscales were similar in the two groups, with the exception of the "Physical activity" subscale, which was significantly lower in the intervention group. Immediately post-intervention and at three months, the intervention group had significantly higher (p<0.001) total and subscale scores, except for "Physical activity," where the initial difference resolved, with both groups reaching similar levels. Over time, the control group showed no significant change in SE, while the intervention group showed a significant increase (p<0.001) immediately post-intervention, maintained at three months. Changes in SE scales differed significantly between groups, with increases in the intervention group and no significant change in the control group (Table 8).

**Table 8:** Changes in DMSES scale and subscales at follow-up Note: the analysis was performed using logarithmic transformations. ^1^P-value for between-group comparisons. ^2^P-value for between-time comparisons after Bonferroni correction. ^3^P-value from repeated measures ANOVA. Differences in change from one measurement to another between groups. DMSES, Diabetes Management Self-Efficacy Scale; SD, standard deviation; SE, self-efficacy

	Group	Before intervention		Immediately after intervention		3 months post-intervention		Change before and immediately after intervention		Change immediately after intervention and 3 months post-intervention		Ρ^3^
		Mean value (SD)	Median (IQR)	Mean value (SD)	Median (IQR)	Mean value (SD)	Median (IQR)	Mean value (SD)	Ρ^2^	Mean value (SD)	Ρ^2^	
SE - diet	Control	29.15 (6.19)	30 (26–33.5)	29.4 (5.87)	30 (26.5–33)	29.13 (5.42)	30 (26–32)	0.25 (1.8)	>0.999	-0.27 (1.96)	>0.999	<0.001
	Intervention	28.93 (4.99)	29 (25–32)	34.18 (3.01)	34 (32–35.5)	34.33 (2.86)	34 (32.5–36)	5.25 (2.94)	<0.001	0.15 (1.89)	>0.999	
	Ρ^1^	0.928		<0.001		<0.001						
SE - treatment	Control	20.72 (1.9)	21 (19–22)	20.92 (1.73)	21 (20–22)	20.67 (1.64)	21 (20–22)	0.2 (1.33)	0.995	-0.25 (1.56)	0.430	<0.001
	Intervention	20.13 (2.38)	21 (18–22)	22.13 (1.23)	22 (21–23)	22.52 (1.1)	23 (22–23)	2 (1.83)	<0.001	0.38 (0.87)	0.086	
	Ρ^1^	0.115		<0.001		<0.001						
SE - medication and foot control	Control	12.83 (1.26)	13 (12–14)	12.65 (1.1)	13 (12–13)	12.52 (1.23)	13 (12–13)	-0.18 (0.85)	0.535	-0.13 (0.93)	0.447	<0.001
	Intervention	12.67 (1.16)	13 (12–14)	13.78 (0.61)	14 (13–14)	13.87 (0.72)	14 (13–14)	1.12 (0.99)	<0.001	0.08 (0.59)	>0.999	
	Ρ^1^	0.499		<0.001		<0.001						
SE - physical activity	Control	11.93 (2.02)	12 (11–13)	11.48 (1.98)	11 (10–13)	11.42 (2.06)	12 (10–13)	-0.45 (0.87)	0.226	-0.07 (1.18)	>0.999	<0.001
	Intervention	10.93 (2.32)	11 (10–13)	11.85 (1.72)	12 (11–13)	11.73 (1.85)	12 (11–13)	0.92 (1.69)	<0.001	-0.12 (0.88)	>0.999	
	Ρ^1^	0.031		0.290		0.382						
SE total (DMSES)	Control	74.63 (9.19)	75 (69–82)	74.45 (8.47)	75.5 (69–80)	73.73 (8.26)	74 (69–81)	-0.18 (2.93)	>0.999	-0.72 (2.88)	0.142	<0.001
	Intervention	72.67 (9.34)	74 (67–80)	81.95 (5.26)	81 (79–85.5)	82.45 (5.38)	83 (79–86.5)	9.28 (5.63)	<0.001	0.5 (2.59)	0.611	
	Ρ^1^	0.251		<0.001		<0.001						

HL (HLS-EU-Q16)

Initially, the two groups had similar HL scores. Immediately post-intervention and at three months, the intervention group had significantly higher HL scores than the control group (p=0.002, p=0.012). Over time, the intervention group showed a significant increase in score (p<0.001), while the control group experienced a significant decrease (p=0.013). At three months, scores in both groups remained similar to post-intervention. The change differed significantly between the groups, with a decrease in the control group and an increase in the intervention group. Table 9 shows patients' HL scores categorized by measure and group.

**Table 9:** HL scale scores by group and measurement Note: the analysis was performed using logarithmic transformations. ^1^P-value for between-group comparisons. ^2^P-value for between-time comparisons after Bonferroni correction. ^3^P-value from repeated measures ANOVA. Differences in change from one measurement to another between groups. HL, health literacy; HLS-EU-Q16, European Health Literacy Survey Questionnaire; SD, standard deviation

Group	HL scores (HLS-EU-Q16)										Ρ^3^
	Before intervention		Immediately after intervention		3 months post-intervention		Change before and immediately after intervention		Change immediately after intervention and 3 months post-intervention		
	Mean value (SD)	Median (IQR)	Mean value (SD)	Median (IQR)	Mean value (SD)	Median (IQR)	Mean value (SD)	Ρ^2^	Mean value (SD)	Ρ^2^	
Control	12.72 (2.33)	13 (11–15)	12.2 (2.07)	12 (11–14)	12.33 (1.95)	12.5 (11–14)	-0.52 (1.2)	0.013	0.13 (0.96)	0.777	<0.001
Intervention	12.63 (2.35)	13 (10.5–15)	13.38 (1.95)	13 (12–15)	13.25 (1.89)	13 (12–15)	0.75 (1.17)	<0.001	-0.13 (1.07)	>0.999	
Ρ^1^	0.857		0.002		0.012						

Initially, the rates of adequate HL were similar in both groups, with 51.7% in the control group and 56.7% in the intervention group. Immediately post-intervention, significant differences emerged, with 45% in the control group and 70% in the intervention group having adequate HL (p=0.012). At three months, the intervention group maintained higher rates of adequate HL. Overall, the control group showed no significant change, while the intervention group had a significant increase (p=0.008) immediately post-intervention, which was sustained for up to three months. Table 10 presents the rates of patients by HL level, categorized by measure and group.

**Table 10:** HL levels categorized by group and measurement ^1^P-value for comparisons between groups via Pearson's χ2 test. ^2^P-value for comparisons between time measurements (McNemar test). HL, health literacy

Group	HL scores (HLS-EU-Q16)										Ρ^3^
	Before intervention		Immediately after intervention		3 months post-intervention		Change before and immediately after intervention		Change immediately after intervention and 3 months post-intervention		
	Mean value (SD)	Median (IQR)	Mean value (SD)	Median (IQR)	Mean value (SD)	Median (IQR)	Mean value (SD)	Ρ^2^	Mean value (SD)	Ρ^2^	
Control	12.72 (2.33)	13 (11–15)	12.2 (2.07)	12 (11–14)	12.33 (1.95)	12.5 (11–14)	-0.52 (1.2)	0.013	0.13 (0.96)	0.777	<0.001
Intervention	12.63 (2.35)	13 (10.5–15)	13.38 (1.95)	13 (12–15)	13.25 (1.89)	13 (12–15)	0.75 (1.17)	<0.001	-0.13 (1.07)	>0.999	
Ρ^1^	0.857		0.002		0.012						

Correlation of HL Changes With Patients’ Characteristics in the Intervention Group

Throughout follow-up, patients under 69 years of age had significantly higher HL scores than those 69 or older, before (p=0.022), immediately post-intervention (p<0.001), and at three months (p<0.001). Before the intervention and at three months, men had significantly higher scores than women (p=0.023, p=0.003). HL scale scores did not differ significantly based on other characteristics at any time point.

In the intervention group, the HL scale scores increased significantly immediately post-intervention in all patients, except for men (p=0.075) and those without complications (p=0.72) and where there was a trend. This increase was maintained at three months. The intervention seemed particularly beneficial for women and those with DM complications, who initially had lower scores. The degree of increase did not vary based on patient characteristics (p>0.05). Table 11 presents the changes in HL according to patient characteristics in the intervention group.

**Table 11:** Changes in HL scale according to patient characteristics in the intervention group Note: analyses were performed using logarithmic transformations. ^1^P-value for between-group comparisons. ^2^P-value for between-time comparisons after Bonferroni correction. ^3^P-value from repeated measures ANOVA. Differences in change from one measurement to another between groups. DM, diabetes mellitus; HL, health literacy; HLS-EU-Q16, European Health Literacy Survey Questionnaire; SD, standard deviation

		HL scores (HLS-EU-Q16)										Ρ^3^
		Before intervention		Immediately after intervention		3 months post-intervention		Change before and immediately after intervention		Change immediately after intervention and 3 months post-intervention		
		Mean value (SD)	Median (IQR)	Mean value (SD)	Median (IQR)	Mean value (SD)	Median (IQR)	Mean value (SD)	Ρ^2^	Mean value (SD)	Ρ^2^	
Age	<69	13.6 (2)	14 (12–15)	14.3 (1.6)	15 (13–16)	14.1 (1.7)	14 (12–15)	0.7 (1.2)	0.015	-0.2 (0.8)	>0.999	0.525
	>=69	11.7 (2.3)	12 (10–14)	12.5 (1.9)	13 (11–14)	12.5 (1.8)	12 (10–14)	0.8 (1.2)	0.001	0 (1.3)	>0.999	
	Ρ^1^	0.002		<0.001		0.001						
Sex	Men	13.4 (2.3)	14 (12–15)	13.9 (1.9)	14 (13–15)	14 (1.7)	14 (12–15)	0.5 (1.1)	0.075	0.1 (1)	>0.999	0.215
	Women	11.9 (2.2)	12 (10–14)	12.9 (1.9)	13 (12–15)	12.5 (1.8)	12 (10–14)	1 (1.3)	<0.001	-0.4 (1.1)	0.253	
	Ρ^1^	0.023		0.066		0.003						
Monthly income in euros	<400	12.5 (2.2)	13 (11–14)	13.5 (1.7)	13 (12–15)	13.3 (1.8)	13 (11–14)	1 (1.3)	0.001	-0.2 (1.3)	>0.999	0.552
	>401	12.7 (2.5)	13.5 (10–15)	13.3 (2.1)	14 (12–15)	13.2 (2)	13.5 (10–15)	0.6 (1.1)	0.008	-0.1 (0.9)	>0.999	
	Ρ^1^	0.794		0.723		0.849						
How many times have you visited your doctor for a DM check-up in the past year?	1-2	13.3 (2.2)	14 (12–15)	14.2 (1.8)	15 (13–16)	14 (1.8)	14 (12–15)	0.9 (1.2)	0.019	-0.2 (0.6)	>0.999	0.948
	3-4	12.4 (2.4)	13 (10–15)	13.1 (1.9)	13 (12–15)	13 (1.9)	13 (10–15)	0.7 (1.2)	0.001	-0.1 (1.2)	>0.999	
	Ρ^1^	0.184		0.056		0.059						
DM medication with insulin	No	12.6 (2.4)	13 (10–15)	13.3 (1.9)	13 (12–15)	13.1 (2)	13 (10–15)	0.7 (1.1)	0.003	-0.2 (1.2)	0.913	0.538
	Yes	12.7 (2.3)	13.5 (11.5–14.5)	13.6 (2)	14 (12.5–15)	13.5 (1.7)	13.5 (11.5–14.5)	0.9 (1.3)	0.003	-0.1 (0.9)	>0.999	
	Ρ^1^	0.830		0.544		0.297						
Diagnosis with any of the following chronic complications of DM?	No	13.2 (2.4)	14 (11–15)	13.7 (2.1)	14 (12–15)	13.5 (2.1)	14 (11–15)	0.5 (1)	0.072	-0.2 (1)	>0.999	0.136
	Yes	12.1 (2.2)	12 (10–14)	13.1 (1.7)	13 (12–14)	13 (1.7)	12 (10–14)	1 (1.3)	<0.001	-0.1 (1.1)	>0.999	
	Ρ^1^	0.107		0.343		0.370						

Correlation Between Changes in SE and HL in the Intervention Group

Mixed linear models were used to examine whether changes in DMSES scores and its subscales were related to changes in HL. Greater increases in HL were associated with larger increases in SE. The results are presented in Table 12.

**Table 12:** Changes in DMSES scores and its subscales in relation to HL +Coefficient of dependence (standard error coefficient). DMSES, Diabetes Management Self-Efficacy Scale; HL, health literacy; HLS-EU-Q16 - European Health Literacy Survey Questionnaire; SE, self-efficacy

	HL scores (HLS-EU-Q16)	
	β (SE)+	P
SE - diet	0.37 (0.06)	<0.001
SE - treatment	0.19 (0.04)	<0.001
SE - medication and foot control	0.26 (0.03)	<0.001
SE - physical activity	0.39 (0.09)	<0.001
SE - total (DMSES)	0.30 (0.04)	<0.001

Multivariate Analyses

Multivariate linear regression was performed with A1C at three months as the dependent variable in all patients. The independent variables included patient demographics (sex, age, income), group allocation, medical history (insulin treatment, complications, frequency of physician visits for DM control in the past year), and baseline HL level. The analysis revealed that insulin use and group assignment were significantly associated with A1C at three months. Specifically, those using insulin had higher A1C values, while participants in the intervention group had significantly lower A1C values at three months. The analysis was conducted using the stepwise inclusion-exclusion method, and the results are presented in Table 13.

**Table 13:** Multivariate analyses of A1C at three months post-intervention in the entire sample Note: logarithmic transformations were used for the analysis. +Coefficient of dependence. ++Coefficient standard error. A1C, glycosylated hemoglobin

		β+	SE++	p-Value
Insulin treatment	No (reference)			
	Yes	0.041	0.010	<0.001
Group	Control (reference)			
	Intervention	-0.046	0.009	<0.001

Multivariate linear regression was performed with HL at three months as the dependent variable. Independent variables included demographics (sex, age, income), group assignment, medical history (insulin treatment, complications, and frequency of doctor visits for DM control in the past year), and baseline HL level. Age (p=0.002), sex (p=0.002), group (p=0.002), and baseline HL (p<0.001) were significantly associated with HL at three months. Participants with adequate baseline HL and those in the intervention group had significantly higher HL scores, while those aged 69 or older and women had lower HL scores. The stepwise inclusion-exclusion method was used, and the results are shown in Table 14.

**Table 14:** Multivariate analyses with HL as the dependent variable at three months post-intervention in the entire sample Note: logarithmic transformations were used for the analysis. +Coefficient of dependence. ++Coefficient standard error. HL, health literacy

		β+	SE++	P
HL levels (before)	Inadequate/problematic (reference)			
	Sufficient	0.080	0.010	<0.001
Age	<69 (reference)			
	≥69	-0.030	0.009	0.002
Sex	Men (reference)			
	Women	-0.030	0.009	0.002
Group	Control (reference)			
	Intervention	0.033	0.009	0.002

Multivariate linear regressions were performed with the SE and its dimensions (excluding the physical activity dimension due to an initial statistically significant difference between the groups) as the dependent variables at three months post-intervention in all patients. Independent variables included demographics (sex, age, income), group assignment, medical history (insulin use, DM complications, frequency of doctor visits for DM control in the past year), and baseline HL level.

The intervention group showed significantly higher scores on the "diet," "treatment," and "medication and foot control," dimensions, as well as the total SE score, at three months compared to the control group. Initial HL levels were significantly associated with the "diet" and "medication and foot control" dimensions, as well as the overall SE score. Specifically, those with adequate baseline HL had significantly higher scores on these scales at three months. Women had significantly lower scores on the "medication and foot control" dimension compared to men. Patients who had visited their doctor three to four times in the past year for DM control had significantly higher scores on the "treatment" dimension than those who visited one to two times. Analyses were performed using the stepwise inclusion-exclusion method, and the results are provided in Table 15.

**Table 15:** Multivariate analyses of SE and its dimensions as the dependent variable at three months post-intervention in the whole sample Note: logarithmic transformations were used for the analyses. +Coefficient of dependence. ++Coefficient standard error. SE, self-efficacy; HL, health literacy; DMSES, Diabetes Management Self-Efficacy Scale

Dependent variables	Independent variables		β+	SE++	P
SE - diet	HL levels (before)	Inadequate/problematic (reference)			
		Sufficient	0.053	0.012	<0.001
	Group	Control (reference)			
		Intervention	0.076	0.012	<0.001
SE - treatment	Group	Control (reference)			
		Intervention	0.025	0.006	<0.001
	How many times in the last year have you visited your doctor to check your diabetes mellitus?	1-2 (reference)			
		3-4	0.013	0.006	0.022
	Sex	Men (reference)			
		Women	-0.017	0.005	0.001
SE - medication and foot control	HL levels (before)	Inadequate/problematic (reference)			
		Sufficient	0.024	0.006	<0.001
	Group	Control (reference)			
		Intervention	0.045	0.006	<0.001
SE - total (DMSES)	HL levels (before)	Inadequate/problematic (reference)			
		Sufficient	0.035	0.007	<0.001
	Group	Control (reference)			
		Intervention	0.049	0.007	<0.001

Mediation of SE in the Relationship Between HL and A1C

The PROCESS procedure was used to test whether SE (measured immediately post-intervention) mediates the relationship between HL (measured immediately post-intervention) and A1C (3 months post-intervention) in the intervention group. The total effect of HL on A1C was statistically significant (β=-0.007, SE=0.002, p=0.003). The direct effect of HL on A1C, considering SE, was not significant (β=-0.001, SE=0.003, p=0.723). However, the indirect effect of SE was significant (β=-0.006, 95% CI=-0.010: -0.003), indicating that SE fully mediates the relationship between HL and A1C.

## Discussion

The results of the study suggest that the intervention had a positive effect on patients in the intervention group. Specifically, it led to improvements in glycemic control outcomes (A1C, FPG and PPG), HL and SE, and weight loss.

The BMI of patients in the intervention group decreased statistically significantly by an average of -0.9 kg/m^2^ three months post-intervention. The findings of similar studies on weight change are inconsistent. A study by Fan et al. [19], which involved individualized education for patients with T2DM, found a statistically significant reduction in BMI (-2.1 kg/m^2^) at six months post-intervention. A systematic review and meta-analysis of educational interventions in patients with T2DM reported a 0.6 kg/m^2^ reduction in BMI [20]. In contrast, a study by Torres et al. [21], using a mixed model of group, home-based education, and telephone intervention, as well as those by Yang et al. [6] and Whittemore et al. [22] involving group education of patients with T2DM found no statistically significant change in BMI at follow-up. The heterogeneity of these results is likely due to variations in training strategies, sociodemographic characteristics of the participants, and the focus of the programs primarily on achieving glycemic goals.

Markers of glycemic control improved at follow-up time regardless of patient characteristics. In the intervention group, A1C decreased by an average of 0.64% at three months post-intervention, and the proportion of patients achieving A1C targets increased from 28.3% to 56.7%. A reduction of 0.5-1.0% in A1C has been associated with a significant decrease in DM-related complications, highlighting the clinical relevance of our findings [6]. Similar improvements have been reported in other studies tailored to patients' HL [23,24]. FPG and PPG also showed significant reductions at three months and an increase in the percentages of patients meeting FPG and PPG targets. These results align with a systematic review of studies from China, which found that HL-tailored educational interventions can improve FPG and PPG in patients with T2DM [24].

HL in our study improved significantly immediately post-intervention and remained stable three months later in the intervention group, suggesting the intervention contributed to this improvement. Studies on the impact of HL-tailored interventions on HL show mixed results. Some systematic reviews and meta-analysis studies in patients with DM report a positive effect [12,24-27], while the study by McGowan et al. found no change following a telephone-based intervention [28].

SE improved significantly in the intervention group across all four dimensions - diet, treatment, medication and foot control, and physical activity - immediately post-intervention, with these gains sustained at three months. This sustained improvement suggests that the HL-tailored intervention effectively empowered patients to adopt and maintain self-care behaviors over time. Similar improvements in SE have been observed in previous studies, with sustained gains reported at 12 months [11] and 6 months [22].

The improvements in HL and SE observed in this study highlight their crucial role in DM management. Given that HL influences patients' ability to understand and apply health-related information and that SE affects their confidence in managing the disease, interventions that enhance both factors can lead to better glycemic control and overall health outcomes. These findings reinforce the importance of incorporating HL- and SE-focused educational strategies into routine DM care.

The relationship between HL, SE, and glycemic goal achievement has been documented in previous cross-sectional studies [8,10]. In our study, SE fully mediated the relationship between HL and A1C, suggesting that HL influences glycemic control primarily by enhancing patients’ confidence in managing their condition. Higher SE has been linked to better adherence to self-care behaviors, including medication adherence, dietary modifications, and physical activity, which are essential for glycemic control. This finding aligns with the intervention study by Kim et al. [29], further supporting the role of SE as a key mechanism in DM self-management.

Our study, along with numerous others [23-27], demonstrates that educational interventions positively impact the effective management of T2DM. Similar programs tailored to patient needs should not only be implemented in research but also be integrated into clinical practice, with ongoing evaluation by healthcare professionals caring for T2DM patients.

Limitations

This study has several limitations that affect its external validity. The primary limitation is that it only assessed results three months post-intervention, without evaluating if or when the positive effects of the intervention diminish. Other limitations include the small sample size (N = 120), the specific population composition (semi-urban, rural), and the fact that the sample was drawn from a single DOC in Greece.

## Conclusions

Home-based educational interventions tailored to the HL of patients with T2DM, with family involvement, appear effective in improving glycemic control, SE, and HL. To maximize long-term benefits, integrating structured patient and family education into primary and community-based healthcare services is crucial. Future efforts should focus on developing sustainable post-hospital care models and adapting educational interventions based on evolving patient needs. Further research is needed to assess the long-term impact of such programs on DM-related complications and healthcare utilization.
